# Null hypothesis significance testing: a short tutorial

**DOI:** 10.12688/f1000research.6963.3

**Published:** 2016-10-10

**Authors:** Cyril Pernet

**Affiliations:** 1 Centre for Clinical Brain Sciences (CCBS), Neuroimaging Sciences, The University of Edinburgh, Edinburgh, UK

**Keywords:** null hypothesis significance testing, tutorial, p-value, reporting, confidence intervals

## Abstract

Although thoroughly criticized, null hypothesis significance testing (NHST) remains the statistical method of choice used to provide evidence for an effect, in biological, biomedical and social sciences. In this short tutorial, I first summarize the concepts behind the method, distinguishing test of significance (Fisher) and test of acceptance (Newman-Pearson) and point to common interpretation errors regarding the p-value. I then present the related concepts of confidence intervals and again point to common interpretation errors. Finally, I discuss what should be reported in which context. The goal is to clarify concepts to avoid interpretation errors and propose reporting practices.

## The Null Hypothesis Significance Testing framework

NHST is a method of statistical inference by which an experimental factor is tested against a hypothesis of no effect or no relationship based on a given observation. The method is a combination of the concepts of significance testing developed by Fisher in 1925 and of acceptance based on critical rejection regions developed by Neyman & Pearson in 1928. In the following I am first presenting each approach, highlighting the key differences and common misconceptions that result from their combination into the NHST framework (for a more mathematical comparison, along with the Bayesian method, see Christensen, 2005). I next present the related concept of confidence intervals. I finish by discussing practical aspects in using NHST and reporting practice.

### Fisher, significance testing, and the p-value

The method developed by ( Fisher, 1934; Fisher, 1955; Fisher, 1959) allows to compute the probability of observing a result at least as extreme as a test statistic (e.g. t value), assuming the null hypothesis of no effect is true. This probability or p-value reflects (1) the conditional probability of achieving the observed outcome or larger: p(Obs≥t|H0), and (2) is therefore a cumulative probability rather than a point estimate. It is equal to the area under the null probability distribution curve from the observed test statistic to the tail of the null distribution ( Turkheimer *et al.*, 2004). The approach proposed is of ‘proof by contradiction’ ( Christensen, 2005), we pose the null model and test if data conform to it.

In practice, it is recommended to set a *level of significance* (a theoretical p-value) that acts as a reference point to identify significant results, that is to identify results that differ from the null-hypothesis of no effect. Fisher recommended using p=0.05 to judge whether an effect is significant or not as it is roughly two standard deviations away from the mean for the normal distribution ( Fisher, 1934 page 45: ‘The value for which p=.05, or 1 in 20, is 1.96 or nearly 2; it is convenient to take this point as a limit in judging whether a deviation is to be considered significant or not’). A key aspect of Fishers’ theory is that only the null-hypothesis is tested, and therefore p-values are meant to be used in a graded manner to decide whether the evidence is worth additional investigation and/or replication ( Fisher, 1971 page 13: ‘it is open to the experimenter to be more or less exacting in respect of the smallness of the probability he would require […]’ and ‘no isolated experiment, however significant in itself, can suffice for the experimental demonstration of any natural phenomenon’). How small the level of significance is, is thus left to researchers.

### What is not a p-value? Common mistakes

The p-value *is not an indication of the strength or magnitude of an effect*. Any interpretation of the p-value in relation to the effect under study (strength, reliability, probability) is wrong, since p-values are conditioned on H0. In addition, while p-values are randomly distributed (if all the assumptions of the test are met) when there is no effect, their distribution depends of both the population effect size and the number of participants, making impossible to infer strength of effect from them.

Similarly, 1-p *is not the probability to replicate an effect*. Often, a small value of p is considered to mean a strong likelihood of getting the same results on another try, but again this cannot be obtained because the p-value is not informative on the effect itself ( Miller, 2009). Because the p-value depends on the number of subjects, it can only be used in high powered studies to interpret results. In low powered studies (typically small number of subjects), the p-value has a large variance across repeated samples, making it unreliable to estimate replication ( Halsey *et al.*, 2015).

A (small) p-value *is not an indication favouring a given hypothesis*. Because a low p-value only indicates a misfit of the null hypothesis to the data, it cannot be taken as evidence in favour of a specific alternative hypothesis more than any other possible alternatives such as measurement error and selection bias ( Gelman, 2013). Some authors have even argued that the more (a priori) implausible the alternative hypothesis, the greater the chance that a finding is a false alarm ( Krzywinski & Altman, 2013; Nuzzo, 2014).

The p-value *is not the probability of the null hypothesis p(H0), of being true,* ( Krzywinski & Altman, 2013). This common misconception arises from a confusion between the probability of an observation given the null p(Obs≥t|H0) and the probability of the null given an observation p(H0|Obs≥t) that is then taken as an indication for p(H0) (see Nickerson, 2000).

### Neyman-Pearson, hypothesis testing, and the α-value

Neyman & Pearson (1933) proposed a framework of statistical inference for applied decision making and quality control. In such framework, two hypotheses are proposed: the null hypothesis of no effect and the alternative hypothesis of an effect, along with a control of the long run probabilities of making errors. The first key concept in this approach, is the establishment of an *alternative hypothesis* along with an a priori effect size. This differs markedly from Fisher who proposed a general approach for scientific inference conditioned on the null hypothesis only. The second key concept is the *control of error rates*. Neyman & Pearson (1928) introduced the notion of critical intervals, therefore dichotomizing the space of possible observations into correct vs. incorrect zones. This dichotomization allows distinguishing correct results (rejecting H0 when there is an effect and not rejecting H0 when there is no effect) from errors (rejecting H0 when there is no effect, the type I error, and not rejecting H0 when there is an effect, the type II error). In this context, alpha is the probability of committing a Type I error in the long run. Alternatively, Beta is the probability of committing a Type II error in the long run.

**Figure 1.**
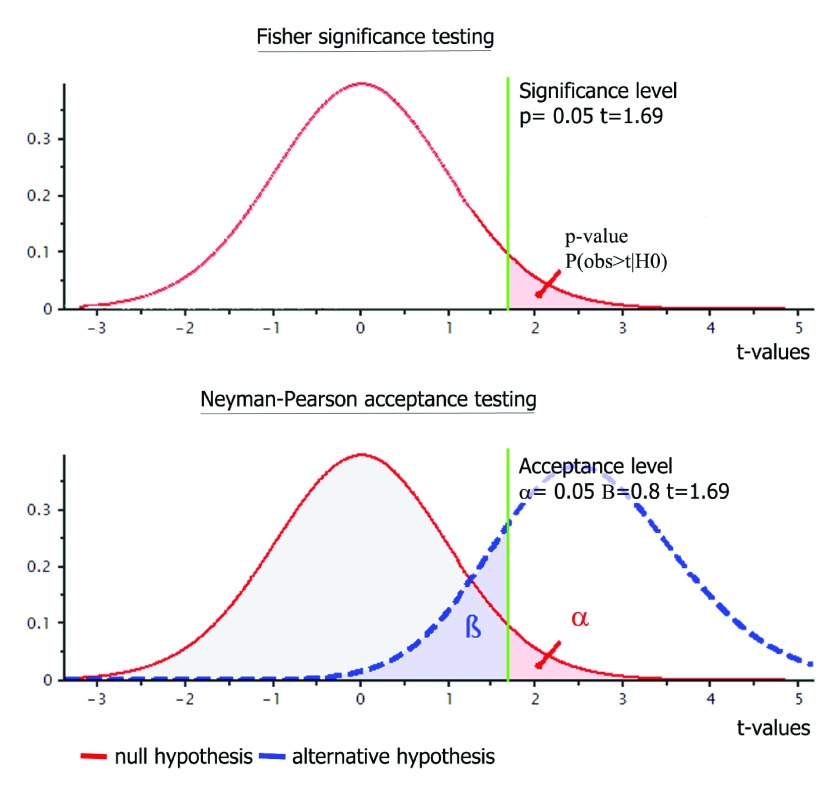
Illustration of the difference between the Fisher and Neyman-Pearson procedures. The figure was prepared with G-power for a one-sided one-sample t-test, with a sample size of 32 subjects, an effect size of 0.45, and error rates alpha=0.049 and beta=0.80. In Fisher’s procedure, only the nil-hypothesis is posed, and the observed p-value is compared to an a priori level of significance. If the observed p-value is below this level (here p=0.05), one rejects H0. In Neyman-Pearson’s procedure, the null and alternative hypotheses are specified along with an a priori level of acceptance. If the observed statistical value is outside the critical region (here [-∞ +1.69]), one rejects H0.

The (theoretical) difference in terms of hypothesis testing between Fisher and Neyman-Pearson is illustrated on Figure 1. In the 1 ^st^ case, we choose a level of significance for observed data of 5%, and compute the p-value. If the p-value is below the level of significance, it is used to reject H0. In the 2 ^nd^ case, we set a critical interval based on the a priori effect size and error rates. If an observed statistic value is below and above the critical values (the bounds of the confidence region), it is deemed significantly different from H0. In the NHST framework, the level of significance is (in practice) assimilated to the alpha level, which appears as a simple decision rule: if the p-value is less or equal to alpha, the null is rejected. It is however a common mistake to assimilate these two concepts. The level of significance set for a given sample is not the same as the frequency of acceptance alpha found on repeated sampling because alpha (a point estimate) is meant to reflect the long run probability whilst the p-value (a cumulative estimate) reflects the current probability ( Fisher, 1955; Hubbard & Bayarri, 2003).

### Acceptance or rejection of H0?

The acceptance level α can also be viewed as the maximum probability that a test statistic falls into the rejection region when the null hypothesis is true ( Johnson, 2013). Therefore, one can only reject the null hypothesis if the test statistics falls into the critical region(s), or fail to reject this hypothesis. In the latter case, all we can say is that no significant effect was observed, but one cannot conclude that the null hypothesis is true. This is another common mistake in using NHST: there is a profound difference between accepting the null hypothesis and simply failing to reject it ( Killeen, 2005). By failing to reject, we simply continue to assume that H0 is true, which implies that one cannot argue against a theory from a non-significant result (absence of evidence is not evidence of absence). To accept the null hypothesis, tests of equivalence ( Walker & Nowacki, 2011) or Bayesian approaches ( Dienes, 2014; Kruschke, 2011) must be used.

### Confidence intervals

Confidence intervals (CI) are builds that fail to cover the true value at a rate of alpha, the Type I error rate ( Morey & Rouder, 2011) and therefore indicate if observed values can be rejected by a (two tailed) test with a given alpha. CI have been advocated as alternatives to p-values because (i) they allow judging the statistical significance and (ii) provide estimates of effect size. Assuming the CI (a)symmetry and width are correct (but see Wilcox, 2012), they also give some indication about the likelihood that a similar value can be observed in future studies. For future studies of the same sample size, 95% CI give about 83% chance of replication success ( Cumming & Maillardet, 2006). If sample sizes however differ between studies, CI do not however warranty any a priori coverage.

Although CI provide more information, they are not less subject to interpretation errors (see Savalei & Dunn, 2015 for a review). The most common mistake is to interpret CI as the probability that a parameter (e.g. the population mean) will fall in that interval X% of the time. The correct interpretation is that, for repeated measurements with the same sample sizes, taken from the same population, X% of times the CI obtained will contain the true parameter value ( Tan & Tan, 2010). The alpha value has the same interpretation as testing against H0, i.e. we accept that 1-alpha CI are wrong in alpha percent of the times in the long run. This implies that CI do not allow to make strong statements about the parameter of interest (e.g. the mean difference) or about H1 ( Hoekstra *et al.*, 2014). To make a statement about the probability of a parameter of interest (e.g. the probability of the mean), Bayesian intervals must be used.

## The (correct) use of NHST

NHST has always been criticized, and yet is still used every day in scientific reports ( Nickerson, 2000). One question to ask oneself is what is the goal of a scientific experiment at hand? If the goal is to establish a discrepancy with the null hypothesis and/or establish a pattern of order, because both requires ruling out equivalence, then NHST is a good tool ( Frick, 1996; Walker & Nowacki, 2011). If the goal is to test the presence of an effect and/or establish some quantitative values related to an effect, then NHST is not the method of choice since testing is conditioned on H0.

While a Bayesian analysis is suited to estimate that the probability that a hypothesis is correct, like NHST, it does not prove a theory on itself, but adds its plausibility ( Lindley, 2000). No matter what testing procedure is used and how strong results are, ( Fisher, 1959 p13) reminds us that ‘ […] no isolated experiment, however significant in itself, can suffice for the experimental demonstration of any natural phenomenon'. Similarly, the recent statement of the American Statistical Association ( Wasserstein & Lazar, 2016) makes it clear that conclusions should be based on the researchers understanding of the problem in context, along with all summary data and tests, and that no single value (being p-values, Bayesian factor or else) can be used support or invalidate a theory.

## What to report and how?

Considering that quantitative reports will always have more information content than binary (significant or not) reports, we can always argue that raw and/or normalized effect size, confidence intervals, or Bayes factor must be reported. Reporting everything can however hinder the communication of the main result(s), and we should aim at giving only the information needed, at least in the core of a manuscript. Here I propose to adopt optimal reporting in the result section to keep the message clear, but have detailed supplementary material. When the hypothesis is about the presence/absence or order of an effect, and providing that a study has sufficient power, NHST is appropriate and it is sufficient to report in the text the actual p-value since it conveys the information needed to rule out equivalence. When the hypothesis and/or the discussion involve some quantitative value, and because p-values do not inform on the effect, it is essential to report on effect sizes ( Lakens, 2013), preferably accompanied with confidence or credible intervals. The reasoning is simply that one cannot predict and/or discuss quantities without accounting for variability. For the reader to understand and fully appreciate the results, nothing else is needed.

Because science progress is obtained by cumulating evidence ( Rosenthal, 1991), scientists should also consider the secondary use of the data. With today’s electronic articles, there are no reasons for not including all of derived data: mean, standard deviations, effect size, CI, Bayes factor should always be included as supplementary tables (or even better also share raw data). It is also essential to report the context in which tests were performed – that is to report all of the tests performed (all t, F, p values) because of the increase type one error rate due to selective reporting (multiple comparisons and p-hacking problems - Ioannidis, 2005). Providing all of this information allows (i) other researchers to directly and effectively compare their results in quantitative terms (replication of effects beyond significance, Open Science Collaboration, 2015), (ii) to compute power to future studies ( Lakens & Evers, 2014), and (iii) to aggregate results for meta-analyses whilst minimizing publication bias ( van Assen *et al.*, 2014).
