# Supplementary material for: Null hypothesis significance testing: a short tutorial
Source: F1000Res. 2016 Oct 10;4:621. Originally published 2015 Aug 25. [Version 3] doi: 10.12688/f1000research.6963.3 (PMC5635437; doi:10.12688/f1000research.6963.3)
Supplement: Peer review history

# Peer review: Referee response for version 3

**DOI:** 10.5256/f1000research.10487.r19282

**Published:** 2017-02-03

**Authors:** Dorothy Vera Margaret Bishop

**Affiliations:** 1 Department of Experimental Psychology, University of Oxford, Oxford, UK

I can see from the history of this paper that the author has already been very responsive to reviewer comments, and that the process of revising has now been quite protracted.

 That makes me reluctant to suggest much more, but I do see potential here for making the paper more impactful. So my overall view is that, once a few typos are fixed (see below), this could be published as is, but I think there is an issue with the potential readership and that further revision could overcome this.

 I suspect my take on this is rather different from other reviewers, as I do not regard myself as a statistics expert, though I am on the more quantitative end of the continuum of psychologists and I try to keep up to date. I think I am quite close to the target readership , insofar as I am someone who was taught about statistics ages ago and uses stats a lot, but never got adequate training in the kinds of topic covered by this paper. The fact that I am aware of controversies around the interpretation of confidence intervals etc is simply because I follow some discussions of this on social media. I am therefore very interested to have a clear account of these issues.

 This paper contains helpful information for someone in this position, but it is not always clear, and I felt the relevance of some of the content was uncertain. So here are some recommendations:

  

1. I wondered about changing the focus slightly and modifying the title to reflect this to say something like: Null hypothesis significance testing: a guide to commonly misunderstood concepts and recommendations for good practice
  As one previous reviewer noted, it’s questionable that there is a need for a tutorial introduction, and the limited length of this article does not lend itself to a full explanation. So it might be better to just focus on explaining as clearly as possible the problems people have had in interpreting key concepts. I think a title that made it clear this was the content would be more appealing than the current one.
2. P 3, col 1, para 3, last sentence. Although statisticians always emphasise the arbitrary nature of p < .05, we all know that in practice authors who use other values are likely to have their analyses queried. I wondered whether it would be useful here to note that in some disciplines different cutoffs are traditional, e.g. particle physics. Or you could cite David Colquhoun’s paper in which he recommends using p < .001 ( http://rsos.royalsocietypublishing.org/content/1/3/140216) - just to be clear that the traditional p < .05 has been challenged.
3. Having read the section on the Fisher approach and Neyman-Pearson approach I felt confused. I have to confess that despite years of doing stats, this distinction had eluded me (which is why I am a good target reader), but I wasn’t really entirely enlightened after reading this. As I understand it, I have been brought up doing null hypothesis testing, so am adopting a Fisher approach. But I also talk about setting alpha to .05, and understand that to come from the Neyman-Pearson approach. If I have understood this correctly, these do amount to the same thing (as the author states, they are assimilated in practice), but we are then told this is a ‘common mistake’. But the explanation of the difference was hard to follow and I found myself wondering whether it would actually make any difference to what I did in practice. In order to understand the last sentence before ‘Acceptance or rejection of H0’ I would need some good analogy. Maybe it would be possible to explain this better with the tried-and-tested example of tossing a coin. So in Fisher approach you do a number of coin tosses to test whether the coin is unbiased (Null hypothesis); you can then work out p as the probability of the null given a specific set of observations, which is the p—value.
  What I can’t work out is how you would explain the alpha from Neyman-Pearson in the same way (though I can see from Figure 1 that with N-P you could test an alternative hypothesis, such as the idea that the coin would be heads 75% of the time).
4. The section on acceptance or rejection of H0 was good, though I found the first sentence a bit opaque and wondered if it could be made clearer. Also I wondered if this rewording would be accurate (as it is clearer to me): instead of:
  ‘By failing to reject, we simply continue to assume that H0 is true, which implies that one cannot….’ have ‘In failing to reject, we do not assume that H0 is true; one cannot argue against a theory from a non-significant result.’
  I felt most readers would be interested to read about tests of equivalence and Bayesian approaches, but many would be unfamiliar with these and might like to see an example of how they work in practice – if space permitted.
5. Confidence intervals: I simply could not understand the first sentence – I wondered what was meant by ‘builds’ here. I understand about difficulties in comparing CI across studies when sample sizes differ, but I did not find the last sentence on p 4 easy to understand.
6. P 5: The sentence starting: ‘The alpha value has the same interpretation’ was also hard to understand, especially the term ‘1-alpha CI’. Here too I felt some concrete illustration might be helpful to the reader. And again, I also found the reference to Bayesian intervals tantalising – I think many readers won’t know how to compute these and something like a figure comparing a traditional CI with a Bayesian interval and giving a source for those who want to read on would be very helpful. The reference to ‘credible intervals’ in the penultimate paragraph is very unclear and needs a supporting reference – most readers will not be familiar with this concept.

 

 Typos etc:

 P 3, col 1, para 2, line 2; “allows us to compute”

 P 3, col 2, para 2, ‘probability of replicating’

 P 3, col 2, para 2, line 4 ‘informative about’

 P 3, col 2, para 4, line 2 delete ‘of’

 P 3, col 2, para 5, line 9 – ‘conditioned’ is either wrong or too technical here: would ‘based’ be acceptable as alternative wording

 P 3, col 2, para 5, line 13 ‘This dichotomisation allows one to distinguish’

 P 3, col 2, para 5, last sentence, delete ‘Alternatively’.

 P 3, col 2, last para line 2 ‘first’

 P 4, col 2, para 2, last sentence is hard to understand; not sure if this is better: ‘If sample sizes differ between studies, the distribution of CIs cannot be specified a priori’

 P 5, col 1, para 2, ‘a pattern of order’ – I did not understand what was meant by this

 P 5, col 1, para 2, last sentence unclear: possible rewording: “If the goal is to test the size of an effect then NHST is not the method of choice, since testing can only reject the null hypothesis.’ (??)

 P 5, col 1, para 3, line 1 delete ‘that’

 P 5, col 1, para 3, line 3 ‘on’ -> ‘by’

 P 5, col 2, para 1, line 4 , rather than ‘Here I propose to adopt’ I suggest ‘I recommend adopting’

 P 5, col 2, para 1, line 13 ‘with’ -> ‘by’

 P 5, col 2, para 1 – recommend deleting last sentence

 P 5, col 2, para 2, line 2 ‘consider’ -> ‘anticipate’

 P 5, col 2, para 2, delete ‘should always be included’

 P 5, col 2, para 2, ‘type one’ -> ‘Type I’

I have read this submission. I believe that I have an appropriate level of expertise to confirm that it is of an acceptable scientific standard, however I have significant reservations, as outlined above.

## References

[1] ColquhounD: An investigation of the false discovery rate and the misinterpretation of p-values. *R Soc Open Sci*.2014;1(3) : 10.1098/rsos.140216 140216 10.1098/rsos.140216 26064558PMC4448847

# Response

**Published:** 2017-09-19

**Authors:** Cyril Pernet

**Affiliations:** The University of Edinburgh, UK

I wondered about changing the focus slightly and modifying the title to reflect this to say something like: Null hypothesis significance testing: a guide to commonly misunderstood concepts and recommendations for good practice

1. As one previous reviewer noted, it’s questionable that there is a need for a tutorial introduction, and the limited length of this article does not lend itself to a full explanation. So it might be better to just focus on explaining as clearly as possible the problems people have had in interpreting key concepts. I think a title that made it clear this was the content would be more appealing than the current one.

 

*Thank you for the suggestion – you indeed saw the intention behind the ‘tutorial’ style of the paper.*


*  *

1. P 3, col 1, para 3, last sentence. Although statisticians always emphasise the arbitrary nature of p < .05, we all know that in practice authors who use other values are likely to have their analyses queried. I wondered whether it would be useful here to note that in some disciplines different cutoffs are traditional, e.g. particle physics. Or you could cite David Colquhoun’s paper in which he recommends using p < .001 ( http://rsos.royalsocietypublishing.org/content/1/3/140216) - just to be clear that the traditional p < .05 has been challenged.

*I have added a sentence on this citing Colquhoun 2014 and the new Benjamin 2017 on using .005.*


*  *

1. Having read the section on the Fisher approach and Neyman-Pearson approach I felt confused. I have to confess that despite years of doing stats, this distinction had eluded me (which is why I am a good target reader), but I wasn’t really entirely enlightened after reading this. As I understand it, I have been brought up doing null hypothesis testing, so am adopting a Fisher approach. But I also talk about setting alpha to .05, and understand that to come from the Neyman-Pearson approach. If I have understood this correctly, these do amount to the same thing (as the author states, they are assimilated in practice), but we are then told this is a ‘common mistake’. But the explanation of the difference was hard to follow and I found myself wondering whether it would actually make any difference to what I did in practice. In order to understand the last sentence before ‘Acceptance or rejection of H0’ I would need some good analogy. Maybe it would be possible to explain this better with the tried-and-tested example of tossing a coin. So in Fisher approach, you do a number of coin tosses to test whether the coin is unbiased (Null hypothesis); you can then work out p as the probability of the null given a specific set of observations, which is the p—value.
  What I can’t work out is how you would explain the alpha from Neyman-Pearson in the same way (though I can see from Figure 1 that with N-P you could test an alternative hypothesis, such as the idea that the coin would be heads 75% of the time).

*I agree that this point is always hard to appreciate, especially because it seems like in practice it makes little difference. I added a paragraph but using reaction times rather than a coin toss – thanks for the suggestion.*


*  *

1. The section on acceptance or rejection of H0 was good, though I found the first sentence a bit opaque and wondered if it could be made clearer. Also I wondered if this rewording would be accurate (as it is clearer to me): instead of:
  ‘By failing to reject, we simply continue to assume that H0 is true, which implies that one cannot….’ have ‘In failing to reject, we do not assume that H0 is true; one cannot argue against a theory from a non-significant result.’
  I felt most readers would be interested to read about tests of equivalence and Bayesian approaches, but many would be unfamiliar with these and might like to see an example of how they work in practice – if space permitted.

*Added an example based on new table 1, following figure 1 – giving CI, equivalence tests and Bayes Factor (with refs to easy to use tools)*


*  *

1. Confidence intervals: I simply could not understand the first sentence – I wondered what was meant by ‘builds’ here. I understand about difficulties in comparing CI across studies when sample sizes differ, but I did not find the last sentence on p 4 easy to understand.

*Changed builds to constructs (this simply means they are something we build) and added that the implication that probability coverage is not warranty when sample size change, is that we cannot compare CI.*


*  *

1. P 5: The sentence starting: ‘The alpha value has the same interpretation’ was also hard to understand, especially the term ‘1-alpha CI’. Here too I felt some concrete illustration might be helpful to the reader. And again, I also found the reference to Bayesian intervals tantalising – I think many readers won’t know how to compute these and something like a figure comparing a traditional CI with a Bayesian interval and giving a source for those who want to read on would be very helpful. The reference to ‘credible intervals’ in the penultimate paragraph is very unclear and needs a supporting reference – most readers will not be familiar with this concept.

*I changed ‘* *i.e. we accept that 1-alpha CI are wrong in alpha percent of the times in the long run’ to ‘, ‘e.g. a 95% CI is wrong in 5% of the times in the long run (i.e. if we repeat the experiment many times).’ – for Bayesian intervals I simply re-cited* Morey & Rouder, 2011.

 Typos

 P 3, col 1, para 2, line 2; “allows us to compute”

 P 3, col 2, para 2, ‘probability of replicating’

 P 3, col 2, para 2, line 4 ‘informative about’

 P 3, col 2, para 4, line 2 delete ‘of’

 P 3, col 2, para 5, line 9 – ‘conditioned’ is either wrong or too technical here: would ‘based’ be acceptable as alternative wording

 P 3, col 2, para 5, line 13 ‘This dichotomisation allows one to distinguish’

 P 3, col 2, para 5, last sentence, delete ‘Alternatively’.

 P 3, col 2, last para line 2 ‘first’

 P 4, col 2, para 2, last sentence is hard to understand; not sure if this is better: ‘If sample sizes differ between studies, the distribution of CIs cannot be specified a priori’

*It is not the CI cannot be specified, it’s that the interval is not predictive of anything anymore! I changed it to ‘If sample* *sizes, however,* *differ between studies, there is no warranty that a CI from one study will be true at the rate alpha in a different study, which implies that* *CI* *cannot be compared across studies at this is rarely the same sample sizes’*

 P 5, col 1, para 2, ‘a pattern of order’ – I did not understand what was meant by this

*I added (i.e. establish that A > B) – we test that conditions are ordered, but without further specification of the probability of that effect nor its size*

 P 5, col 1, para 2, last sentence unclear: possible rewording: “If the goal is to test the size of an effect then NHST is not the method of choice, since testing can only reject the null hypothesis.’ (??)

*Yes it works – thx*

 P 5, col 1, para 3, line 1 delete ‘that’

 P 5, col 1, para 3, line 3 ‘on’ -> ‘by’

 P 5, col 2, para 1, line 4 , rather than ‘Here I propose to adopt’ I suggest ‘I recommend adopting’

 P 5, col 2, para 1, line 13 ‘with’ -> ‘by’

 P 5, col 2, para 1 – recommend deleting last sentence

 P 5, col 2, para 2, line 2 ‘consider’ -> ‘anticipate’

 P 5, col 2, para 2, delete ‘should always be included’

 P 5, col 2, para 2, ‘type one’ -> ‘Type I’ 

*Typos fixed, and suggestions accepted – thanks for that.*

# Peer review: Referee response for version 3

**DOI:** 10.5256/f1000research.10487.r17400

**Published:** 2016-11-03

**Authors:** Stephen J. Senn

**Affiliations:** 1 Luxembourg Institute of Health, Strassen, L-1445, Luxembourg

The revisions are OK for me, and I have changed my status to Approved.

I have read this submission. I believe that I have an appropriate level of expertise to confirm that it is of an acceptable scientific standard.

# Peer review: Referee response for version 2

**DOI:** 10.5256/f1000research.9903.r16257

**Published:** 2016-09-28

**Authors:** Stephen J. Senn

**Affiliations:** 1 Luxembourg Institute of Health, Strassen, L-1445, Luxembourg

On the whole I think that this article is reasonable, my main reservation being that I have my doubts on whether the literature needs yet another tutorial on this subject.

 A further reservation I have is that the author, following others, stresses what in my mind is a relatively unimportant distinction between the Fisherian and Neyman-Pearson (NP) approaches. The distinction stressed by many is that the NP approach leads to a dichotomy accept/reject based on probabilities established in advance, whereas the Fisherian approach uses tail area probabilities calculated from the observed statistic. I see this as being unimportant and not even true. Unless one considers that the person carrying out a hypothesis test (original tester) is mandated to come to a conclusion on behalf of all scientific posterity, then one must accept that any remote scientist can come to his or her conclusion depending on the personal type I error favoured. To operate the results of an NP test carried out by the original tester, the remote scientist then needs to know the p-value. The type I error rate is then compared to this to come to a personal accept or reject decision (1). In fact Lehmann (2), who was an important developer of and proponent of the NP system, describes exactly this approach as being good practice. (See Testing Statistical Hypotheses, 2nd edition P70). Thus using tail-area probabilities calculated from the observed statistics does not constitute an operational difference between the two systems.

 A more important distinction between the Fisherian and NP systems is that the former does not use alternative hypotheses(3). Fisher's opinion was that the null hypothesis was more primitive than the test statistic but that the test statistic was more primitive than the alternative hypothesis. Thus, alternative hypotheses could not be used to justify choice of test statistic. Only experience could do that.

 Further distinctions between the NP and Fisherian approach are to do with conditioning and whether a null hypothesis can ever be accepted.

 I have one minor quibble about terminology. As far as I can see, the author uses the usual term 'null hypothesis' and the eccentric term 'nil hypothesis' interchangeably. It would be simpler if the latter were abandoned.

I have read this submission. I believe that I have an appropriate level of expertise to confirm that it is of an acceptable scientific standard, however I have significant reservations, as outlined above.

## References

[1] SennS: A comment on replication,p-values and evidence S.N.Goodman,Statistics in Medicine 1992;11:875-879. *Statistics in Medicine*.2002;21(16) : 10.1002/sim.1072 2437-2444 10.1002/sim.1072 12210627

[2] LehmannE L: Testing Statistical Hypotheses, 2nd edition. *Chapman and Hall*.1993;

[3] SennS: You may believe you are a Bayesian but you are probably wrong. *RMM*.2011;2:41-66 Reference source

# Peer review: Referee response for version 1

**DOI:** 10.5256/f1000research.7499.r11036

**Published:** 2015-11-10

**Authors:** Marcel ALM van Assen

**Affiliations:** 1 Department of Methodology and Statistics, Tilburgh University, Tilburg, Netherlands

Null hypothesis significance testing (NHST) is a difficult topic, with misunderstandings arising easily. Many texts, including basic statistics books, deal with the topic, and attempt to explain it to students and anyone else interested. I would refer to a good basic text book, for a detailed explanation of NHST, or to a specialized article when wishing an explaining the background of NHST. So, what is the added value of a new text on NHST? In any case, the added value should be described at the start of this text. Moreover, the topic is so delicate and difficult that errors, misinterpretations, and disagreements are easy. I attempted to show this by giving comments to many sentences in the text.

Abstract: “null hypothesis significance testing is the statistical method of choice in biological, biomedical and social sciences to investigate if an effect is likely”. No, NHST is the method to test the hypothesis of no effect.

Intro: “Null hypothesis significance testing (NHST) is a method of statistical inference by which an observation is tested against a hypothesis of no effect or no relationship.” What is an ‘observation’? NHST is difficult to describe in one sentence, particularly here. I would skip this sentence entirely, here.

Section on Fisher; also explain the one-tailed test.

Section on Fisher; p(Obs|H0) does not reflect the verbal definition (the ‘or more extreme’ part).

Section on Fisher; use a reference and citation to Fisher’s interpretation of the p-value

Section on Fisher; “This was however only intended to be used as an indication that there is something in the data that deserves further investigation. The reason for this is that only H0 is tested whilst the effect under study is not itself being investigated.” First sentence, can you give a reference? Many people say a lot about Fisher’s intentions, but the good man is dead and cannot reply… Second sentence is a bit awkward, because the effect is investigated in a way, by testing the H0.

Section on p-value; Layout and structure can be improved greatly, by first again stating what the p-value is, and then statement by statement, what it is not, using separate lines for each statement. Consider adding that the p-value is randomly distributed under H0 (if all the assumptions of the test are met), and that under H1 the p-value is a function of population effect size and N; the larger each is, the smaller the p-value generally is.

Skip the sentence “If there is no effect, we should replicate the absence of effect with a probability equal to 1-p”. Not insightful, and you did not discuss the concept ‘replicate’ (and do not need to).

Skip the sentence “The total probability of false positives can also be obtained by aggregating results ( Ioannidis, 2005).” Not strongly related to p-values, and introduces unnecessary concepts ‘false positives’ (perhaps later useful) and ‘aggregation’.

Consider deleting; “If there is an effect however, the probability to replicate is a function of the (unknown) population effect size with no good way to know this from a single experiment ( Killeen, 2005).”

The following sentence; “ Finally, a (small) p-value *is not an indication favouring a hypothesis*. A low p-value indicates a misfit of the null hypothesis to the data and cannot be taken as evidence in favour of a specific alternative hypothesis more than any other possible alternatives such as measurement error and selection bias ( Gelman, 2013).” is surely not mainstream thinking about NHST; I would surely delete that sentence. In NHST, a p-value is used for testing the H0. Why did you not yet discuss significance level? Yes, before discussing what is not a p-value, I would explain NHST (i.e., what it is and how it is used). 

Also the next sentence “The more (a priori) implausible the alternative hypothesis, the greater the chance that a finding is a false alarm ( Krzywinski & Altman, 2013; Nuzzo, 2014).“ is not fully clear to me. This is a Bayesian statement. In NHST, no likelihoods are attributed to hypotheses; the reasoning is “IF H0 is true, then…”.

Last sentence: “As Nickerson (2000) puts it ‘theory corroboration requires the testing of multiple predictions because the chance of getting statistically significant results for the wrong reasons in any given case is high’.” What is relation of this sentence to the contents of this section, precisely?

Next section: “For instance, we can estimate that the probability of a given F value to be in the critical interval [+2 +∞] is less than 5%” This depends on the degrees of freedom.

“When there is no effect (H0 is true), the erroneous rejection of H0 is known as type I error and is equal to the p-value.” Strange sentence. The Type I error is the probability of erroneously rejecting the H0 (so, when it is true). The p-value is … well, you explained it before; it surely does not equal the Type I error.

Consider adding a figure explaining the distinction between Fisher’s logic and that of Neyman and Pearson.

“When the test statistics falls outside the critical region(s)” What is outside?

“There is a profound difference between accepting the null hypothesis and simply failing to reject it ( Killeen, 2005)” I agree with you, but perhaps you may add that some statisticians simply define “accept H0’” as obtaining a p-value larger than the significance level. Did you already discuss the significance level, and it’s mostly used values?

“To accept or reject equally the null hypothesis, Bayesian approaches ( Dienes, 2014; Kruschke, 2011) or confidence intervals must be used.” Is ‘reject equally’ appropriate English? Also using Cis, one cannot accept the H0.

Do you start discussing alpha only in the context of Cis?

“CI also indicates the precision of the estimate of effect size, but unless using a percentile bootstrap approach, they require assumptions about distributions which can lead to serious biases in particular regarding the symmetry and width of the intervals ( Wilcox, 2012).” Too difficult, using new concepts. Consider deleting.

“Assuming the CI (a)symmetry and width are correct, this gives some indication about the likelihood that a similar value can be observed in future studies, with 95% CI giving about 83% chance of replication success ( Lakens & Evers, 2014).” This statement is, in general, completely false. It very much depends on the sample sizes of both studies. If the replication study has a much, much, much larger N, then the probability that the original CI will contain the effect size of the replication approaches (1-alpha)*100%. If the original study has a much, much, much larger N, then the probability that the original Ci will contain the effect size of the replication study approaches 0%.

“Finally, contrary to p-values, CI can be used to accept H0. Typically, if a CI includes 0, we cannot reject H0. If a critical null region is specified rather than a single point estimate, for instance [-2 +2] and the CI is included within the critical null region, then H0 can be accepted. Importantly, the critical region must be specified a priori and cannot be determined from the data themselves.” No. H0 cannot be accepted with Cis.

“The (posterior) probability of an effect can however not be obtained using a frequentist framework.” Frequentist framework? You did not discuss that, yet.

“X% of times the CI obtained will contain the same parameter value”. The same? True, you mean?

“e.g. X% of the times the CI contains the same mean” I do not understand; which mean?

“The alpha value has the same interpretation as when using H0, i.e. we accept that 1-alpha CI are wrong in alpha percent of the times. “ What do you mean, CI are wrong? Consider rephrasing.

“To make a statement about the probability of a parameter of interest, likelihood intervals (maximum likelihood) and credibility intervals (Bayes) are better suited.” ML gives the likelihood of the data given the parameter, not the other way around.

“Many of the disagreements are not on the method itself but on its use.” Bayesians may disagree.

“If the goal is to establish the likelihood of an effect and/or establish a pattern of order, because both requires ruling out equivalence, then NHST is a good tool ( Frick, 1996)” NHST does not provide evidence on the likelihood of an effect.

“If the goal is to establish some quantitative values, then NHST is not the method of choice.” P-values are also quantitative… this is not a precise sentence. And NHST may be used in combination with effect size estimation (this is even recommended by, e.g., the American Psychological Association (APA)).

“Because results are conditioned on H0, NHST cannot be used to establish beliefs.” It can reinforce some beliefs, e.g., if H0 or any other hypothesis, is true.

“To estimate the probability of a hypothesis, a Bayesian analysis is a better alternative.” It is the only alternative?

“Note however that even when a specific quantitative prediction from a hypothesis is shown to be true (typically testing H1 using Bayes), it does not prove the hypothesis itself, it only adds to its plausibility.” How can we *show* something is true?

I do not agree on the contents of the last section on ‘minimal reporting’. I prefer ‘optimal reporting’ instead, i.e., the reporting the information that is essential to the interpretation of the result, to any ready, which may have other goals than the writer of the article. This reporting includes, for sure, an estimate of effect size, and preferably a confidence interval, which is in line with recommendations of the APA.

I have read this submission. I believe that I have an appropriate level of expertise to state that I do not consider it to be of an acceptable scientific standard, for reasons outlined above.

# Response

**Published:** 2016-07-06

**Authors:** Cyril Pernet

**Affiliations:** The University of Edinburgh, UK

- *Null hypothesis significance testing (NHST) is a difficult topic, with misunderstandings arising easily. Many texts, including basic statistics books, deal with the topic, and attempt to explain it to students and anyone else interested. I would refer to a good basic text book, for a detailed explanation of NHST, or to a specialized article when wishing an explaining the background of NHST. So, what is the added value of a new text on NHST? In any case, the added value should be described at the start of this text. Moreover, the topic is so delicate and difficult that errors, misinterpretations, and disagreements are easy. I attempted to show this by giving comments to many sentences in the text.*
  The idea of this short review was to point to common interpretation errors (stressing again and again that we are under H0) being in using p-values or CI, and also proposing reporting practices to avoid bias. This is now stated at the end of abstract.
  Regarding text books, it is clear that many fail to clearly distinguish Fisher/Pearson/NHST, see Glinet et al (2012) J. Exp Education 71, 83-92. If you have 1 or 2 in mind that you know to be good, I’m happy to include them.
- *Abstract: “null hypothesis significance testing is the statistical method of choice in biological, biomedical and social sciences to investigate if an effect is likely”. No, NHST is the method to test the hypothesis of no effect.*
  I agree – yet people use it to investigate (not test) if an effect is likely. The issue here is wording. What about adding this distinction at the end of the sentence?: ‘null hypothesis significance testing is the statistical method of choice in biological, biomedical and social sciences used to investigate if an effect is likely, even though it actually tests for the hypothesis of no effect’.
- *Intro: “Null hypothesis significance testing (NHST) is a method of statistical inference by which an observation is tested against a hypothesis of no effect or no relationship.” What is an ‘observation’? NHST is difficult to describe in one sentence, particularly here. I would skip this sentence entirely, here.*
  I think a definition is needed, as it offers a starting point. What about the following: ‘NHST is a method of statistical inference by which an experimental factor is tested against a hypothesis of no effect or no relationship based on a given observation’
- *Section on Fisher; also explain the one-tailed test.*
*Section on Fisher; p(Obs|H0) does not reflect the verbal definition (the ‘or more extreme’ part).*
*Section on Fisher; use a reference and citation to Fisher’s interpretation of the p-value*
*Section on Fisher; “This was however only intended to be used as an indication that there is something in the data that deserves further investigation. The reason for this is that only H0 is tested whilst the effect under study is not itself being investigated.” First sentence, can you give a reference? Many people say a lot about Fisher’s intentions, but the good man is dead and cannot reply… Second sentence is a bit awkward, because the effect is investigated in a way, by testing the H0.*
  The section on Fisher has been modified (more or less) as suggested: (1) avoiding talking about one or two tailed tests (2) updating for p(Obs≥t|H0) and (3) referring to Fisher more explicitly (ie pages from articles and book) ; I cannot tell his intentions but these quotes leave little space to alternative interpretations.
- *Section on p-value; Layout and structure can be improved greatly, by first again stating what the p-value is, and then statement by statement, what it is not, using separate lines for each statement. Consider adding that the p-value is randomly distributed under H0 (if all the assumptions of the test are met), and that under H1 the p-value is a function of population effect size and N; the larger each is, the smaller the p-value generally is.*
  Done
- *Skip the sentence “If there is no effect, we should replicate the absence of effect with a probability equal to 1-p”. Not insightful, and you did not discuss the concept ‘replicate’ (and do not need to).*
  Done
- *Skip the sentence “The total probability of false positives can also be obtained by aggregating results (* *Ioannidis, 2005* *).” Not strongly related to p-values, and introduces unnecessary concepts ‘false positives’ (perhaps later useful) and ‘aggregation’.*
  Done
- *Consider deleting; “If there is an effect however, the probability to replicate is a function of the (unknown) population effect size with no good way to know this from a single experiment (* *Killeen, 2005* *).”*
  Done
- *The following sentence; “ Finally, a (small) p-value is not an indication favouring a hypothesis. A low p-value indicates a misfit of the null hypothesis to the data and cannot be taken as evidence in favour of a specific alternative hypothesis more than any other possible alternatives such as measurement error and selection bias ( Gelman, 2013).” is surely not mainstream thinking about NHST; I would surely delete that sentence. In NHST, a p-value is used for testing the H0. Why did you not yet discuss significance level? Yes, before discussing what is not a p-value, I would explain NHST (i.e., what it is and how it is used). *
*Also the next sentence “The more (a priori) implausible the alternative hypothesis, the greater the chance that a finding is a false alarm ( Krzywinski & Altman, 2013; Nuzzo, 2014).“ is not fully clear to me. This is a Bayesian statement. In NHST, no likelihoods are attributed to hypotheses; the reasoning is “IF H0 is true, then…”.*
  The reasoning here is as you state yourself, part 1: ‘a p-value is used for testing the H0; and part 2: ‘no likelihoods are attributed to hypotheses’ it follows we cannot favour a hypothesis. It might seems contentious but this is the case that all we can is to reject the null – how could we favour a specific alternative hypothesis from there? This is explored further down the manuscript (and I now point to that) – note that we do not need to be Bayesian to favour a specific H1, all I’m saying is this cannot be attained with a p-value.
- *Last sentence: “As* *Nickerson (2000)* *puts it ‘theory corroboration requires the testing of multiple predictions because the chance of getting statistically significant results for the wrong reasons in any given case is high’.” What is relation of this sentence to the contents of this section, precisely?*
  The point was to emphasise that a p value is not there to tell us a given H1 is true and can only be achieved through multiple predictions and experiments. I deleted it for clarity.
- *Next section: “For instance, we can estimate that the probability of a given F value to be in the critical interval [+2 +∞] is less than 5%” This depends on the degrees of freedom.*
  This sentence has been removed
- *“When there is no effect (H0 is true), the erroneous rejection of H0 is known as type I error and is equal to the p-value.” Strange sentence. The Type I error is the probability of erroneously rejecting the H0 (so, when it is true). The p-value is … well, you explained it before; it surely does not equal the Type I error.*
  Indeed, you are right and I have modified the text accordingly. When there is no effect (H0 is true), the erroneous rejection of H0 is known as type 1 error. Importantly, the type 1 error rate, or alpha value is determined a priori. It is a common mistake but the level of significance (for a given sample) is not the same as the frequency of acceptance alpha found on repeated sampling (Fisher, 1955).
- *Consider adding a figure explaining the distinction between Fisher’s logic and that of Neyman and Pearson.*
  A figure is now presented – with levels of acceptance, critical region, level of significance and p-value.
- “ *When the test statistics falls outside the critical region(s)” What is outside?*
**
* “There is a profound difference between accepting the null hypothesis and simply failing to reject it ( Killeen, 2005)” I agree with you, but perhaps you may add that some statisticians simply define “accept H0’” as obtaining a p-value larger than the significance level. Did you already discuss the significance level, and it’s mostly used values?*
**
* “To accept or reject equally the null hypothesis, Bayesian approaches ( Dienes, 2014; Kruschke, 2011) or confidence intervals must be used.” Is ‘reject equally’ appropriate English? Also using Cis, one cannot accept the H0.*
  I should have clarified further here – as I was having in mind tests of equivalence. To clarify, I simply states now: ‘To accept the null hypothesis, tests of equivalence or Bayesian approaches must be used.’
- *Do you start discussing alpha only in the context of Cis?*
  It is now presented in the paragraph before.
- *“CI also indicates the precision of the estimate of effect size, but unless using a percentile bootstrap approach, they require assumptions about distributions which can lead to serious biases in particular regarding the symmetry and width of the intervals ( Wilcox, 2012).” Too difficult, using new concepts. Consider deleting.*
  Done
- “Assuming the CI (a)symmetry and width are correct, this gives some indication about the likelihood that a similar value can be observed in future studies, with 95% CI giving about 83% chance of replication success ( Lakens & Evers, 2014).” This statement is, in general, completely false. It very much depends on the sample sizes of both studies. If the replication study has a much, much, much larger N, then the probability that the original CI will contain the effect size of the replication approaches (1-alpha)*100%. If the original study has a much, much, much larger N, then the probability that the original Ci will contain the effect size of the replication study approaches 0%.
  Yes, you are right, I completely overlooked this problem. The corrected sentence (with more accurate ref) is now *“Assuming the CI (a)symmetry and width are correct, this gives some indication about the likelihood that a similar value can be observed in future studies. For future studies of the same sample size, 95% CI giving about 83% chance of replication success (Cumming and Mallardet, 2006). If sample sizes differ between studies, CI do not however warranty any a priori coverage”. *
- *“Finally, contrary to p-values, CI can be used to accept H0. Typically, if a CI includes 0, we cannot reject H0. If a critical null region is specified rather than a single point estimate, for instance [-2 +2] and the CI is included within the critical null region, then H0 can be accepted. Importantly, the critical region must be specified a priori and cannot be determined from the data themselves.” No. H0 cannot be accepted with Cis.*
  Again, I had in mind equivalence testing, but in both cases you are right we can only reject and I therefore removed that sentence.
- *“The (posterior) probability of an effect can however not be obtained using a frequentist framework.” Frequentist framework? You did not discuss that, yet.*
  Removed
- *“X% of times the CI obtained will contain the same parameter value”. The same? True, you mean?*
**
* “e.g. X% of the times the CI contains the same mean” I do not understand; which mean?*
**
* “The alpha value has the same interpretation as when using H0, i.e. we accept that 1-alpha CI are wrong in alpha percent of the times. “ What do you mean, CI are wrong? Consider rephrasing.*
**
* “To make a statement about the probability of a parameter of interest, likelihood intervals (maximum likelihood) and credibility intervals (Bayes) are better suited.” ML gives the likelihood of the data given the parameter, not the other way around.*
  corrected
- “ *Many of the disagreements are not on the method itself but on its use.” Bayesians may disagree.*
  removed
- *“If the goal is to establish the likelihood of an effect and/or establish a pattern of order, because both requires ruling out equivalence, then NHST is a good tool ( Frick, 1996)” NHST does not provide evidence on the likelihood of an effect.*
  “If the goal is to establish some quantitative values, then NHST is not the method of choice.” P-values are also quantitative… this is not a precise sentence. And NHST may be used in combination with effect size estimation (this is even recommended by, e.g., the American Psychological Association (APA)).
  Yes, p-values must be interpreted in context with effect size, but this is not what people do. The point here is to be pragmatic, does and don’t. The sentence was changed.
- *“Because results are conditioned on H0, NHST cannot be used to establish beliefs.” It can reinforce some beliefs, e.g., if H0 or any other hypothesis, is true.*
**
* “To estimate the probability of a hypothesis, a Bayesian analysis is a better alternative.” It is the only alternative?*
  Not for testing, but for probability, I am not aware of anything else.
- *“Note however that even when a specific quantitative prediction from a hypothesis is shown to be true (typically testing H1 using Bayes), it does not prove the hypothesis itself, it only adds to its plausibility.” How can we show something is true?*
  Cumulative evidence is, in my opinion, the only way to show it. Even in hard science like physics multiple experiments. In the recent CERN study on finding Higgs bosons, 2 different and complementary experiments ran in parallel – and the cumulative evidence was taken as a proof of the true existence of Higgs bosons.

# Peer review: Referee response for version 1

**DOI:** 10.5256/f1000research.7499.r10159

**Published:** 2015-10-30

**Authors:** Daniel Lakens

**Affiliations:** 1 School of Innovation Sciences, Eindhoven University of Technology, Eindhoven, Netherlands

I appreciate the author's attempt to write a short tutorial on NHST. Many people don't know how to use it, so attempts to educate people are always worthwhile. However, I don't think the current article reaches it's aim. For one, I think it might be practically impossible to explain a lot in such an ultra short paper - every section would require more than 2 pages to explain, and there are many sections. Furthermore, there are some excellent overviews, which, although more extensive, are also much clearer (e.g., Nickerson, 2000). Finally, I found many statements to be unclear, and perhaps even incorrect (noted below). Because there is nothing worse than creating more confusion on such a topic, I have extremely high standards before I think such a short primer should be indexed. I note some examples of unclear or incorrect statements below. I'm sorry I can't make a more positive recommendation.

“investigate if an effect is likely” – ambiguous statement. I think you mean, whether the observed DATA is probable, assuming there is no effect?

The Fisher (1959) reference is not correct – Fischer developed his method much earlier.

“This p-value thus reflects the conditional probability of achieving the observed outcome or larger, p(Obs|H0)” – please add 'assuming the null-hypothesis is true'.

“p(Obs|H0)” – explain this notation for novices.

“Following Fisher, the smaller the p-value, the greater the likelihood that the null hypothesis is false.”  This is wrong, and any statement about this needs to be much more precise. I would suggest direct quotes.

“there is something in the data that deserves further investigation” –unclear sentence.

“The reason for this” – unclear what ‘this’ refers to.

“ *not the probability of the null hypothesis of being true, p(H0)” *– second of can be removed?

“Any interpretation of the p-value in relation to the effect under study (strength, reliability, probability) is indeed

wrong, since the p-value is conditioned on H0”  - incorrect. A big problem is that it depends on the sample size, and that the probability of a theory depends on the prior.

“If there is no effect, we should replicate the absence of effect with a probability equal to 1-p.” I don’t understand this, but I think it is incorrect.

“The total probability of false positives can also be obtained by aggregating results (Ioannidis, 2005).” Unclear, and probably incorrect.

“By failing to reject, we simply continue to assume that H0 is true, which implies that one cannot, from a nonsignificant result, argue against a theory” – according to which theory? From a NP perspective, you can ACT as if the theory is false.

“(Lakens & Evers, 2014”) – we are not the original source, which should be cited instead.

“ Typically, if a CI includes 0, we cannot reject H0.”  - when would this not be the case? This assumes a CI of 1-alpha.

“If a critical null region is specified rather than a single point estimate, for instance [-2 +2] and the CI is included within the critical null region, then H0 can be accepted.” – you mean practically, or formally? I’m pretty sure only the former.

The section on ****‘The (correct) use of NHST’ seems to conclude only Bayesian statistics should be used. I don’t really agree.

**“**we can always argue that effect size, power, etc. must be reported.” – which power? Post-hoc power? Surely not? Other types are unknown. So what do you mean?

The recommendation on what to report remains vague, and it is unclear why what should be reported.

I have read this submission. I believe that I have an appropriate level of expertise to state that I do not consider it to be of an acceptable scientific standard, for reasons outlined above.

# Response

**Published:** 2016-07-06

**Authors:** Cyril Pernet

**Affiliations:** The University of Edinburgh, UK

- *“investigate if an effect is likely” – ambiguous statement. I think you mean, whether the observed DATA is probable, assuming there is no effect?*
  This sentence was changed, following as well the other reviewer, to ‘null hypothesis significance testing is the statistical method of choice in biological, biomedical and social sciences to investigate if an effect is likely, even though it actually tests whether the observed data are probable, assuming there is no effect’
- *The Fisher (1959) reference is not correct – Fischer developed his method much earlier.*
  Changed, refers to Fisher 1925
- *“This p-value thus reflects the conditional probability of achieving the observed outcome or larger, p(Obs|H0)” – please add 'assuming the null-hypothesis is true'. “p(Obs|H0)” – explain this notation for novices.*
  I changed a little the sentence structure, which should make explicit that this is the condition probability.
- *“Following Fisher, the smaller the p-value, the greater the likelihood that the null hypothesis is false.”  This is wrong, and any statement about this needs to be much more precise. I would suggest direct quotes.*
  This sentence has been removed
- *“there is something in the data that deserves further investigation” –unclear sentence. “The reason for this” – unclear what ‘this’ refers to.*
  This has been changed to ‘[…] to decide whether the evidence is worth additional investigation and/or replication (Fisher, 1971 p13)’
- *“not the probability of the null hypothesis of being true, p(H0)” – second of can be removed?*
  my mistake – the sentence structure is now ‘ *not the probability of the null hypothesis p(H0), of being true,’ ; hope this makes more sense (and this way refers back to p(Obs>t|H0)*
- *“Any interpretation of the p-value in relation to the effect under study (strength, reliability, probability) is indeed wrong, since the p-value is conditioned on H0”  - incorrect. A big problem is that it depends on the sample size, and that the probability of a theory depends on the prior.*
  Fair enough – my point was to stress the fact that p value and effect size or H1 have very little in common, but yes that the part in common has to do with sample size. I left the conditioning on H0 but also point out the dependency on sample size.
- *“If there is no effect, we should replicate the absence of effect with a probability equal to 1-p.” I don’t understand this, but I think it is incorrect.*
  Removed
- *“The total probability of false positives can also be obtained by aggregating results (Ioannidis, 2005).” Unclear, and probably incorrect.*
  Removed
- *“By failing to reject, we simply continue to assume that H0 is true, which implies that one cannot, from a nonsignificant result, argue against a theory” – according to which theory? From a NP perspective, you can ACT as if the theory is false.*
  The whole paragraph was changed to reflect a more philosophical take on scientific induction/reasoning. I hope this is clearer.
- *“(Lakens & Evers, 2014”) – we are not the original source, which should be cited instead.*
  done
- *“ Typically, if a CI includes 0, we cannot reject H0.”  - when would this not be the case? This assumes a CI of 1-alpha. “If a critical null region is specified rather than a single point estimate, for instance [-2 +2] and the CI is included within the critical null region, then H0 can be accepted.” – you mean practically, or formally? I’m pretty sure only the former.*
  Changed to refer to equivalence testing
- *The section on ‘The (correct) use of NHST’ seems to conclude only Bayesian statistics should be used. I don’t really agree.*
  I rewrote this, as to show frequentist analysis can be used  - I’m trying to sell Bayes more than any other approach.
- ***“**we can always argue that effect size, power, etc. must be reported.” – which power? Post-hoc power? Surely not? Other types are unknown. So what do you mean? The recommendation on what to report remains vague, and it is unclear why what should be reported.*
  I’m arguing we should report it all, that’s why there is no exhausting list – I can if needed.
